# An Update on Mesenchymal Stem Cell-Centered Therapies in Temporomandibular Joint Osteoarthritis

**DOI:** 10.1155/2021/6619527

**Published:** 2021-04-01

**Authors:** Yifan Zhao, Liang Xie

**Affiliations:** State Key Laboratory of Oral Diseases, National Clinical Research Center for Oral Diseases, Chinese Academy of Medical Sciences Research Unit of Oral Carcinogenesis and Management, West China Hospital of Stomatology, Sichuan University, Chengdu, Sichuan, China

## Abstract

Temporomandibular joint osteoarthritis (TMJOA) is a degenerative disease characterized by cartilage degeneration, disrupted subchondral bone remodeling, and synovitis, seriously affecting the quality of life of patients with chronic pain and functional disabilities. Current treatments for TMJOA are mainly symptomatic therapies without reliable long-term efficacy, due to the limited self-renewal capability of the condyle and the poorly elucidated pathogenesis of TMJOA. Recently, there has been increased interest in cellular therapies for osteoarthritis and TMJ regeneration. Mesenchymal stem cells (MSCs), self-renewing and multipotent progenitor cells, play a promising role in TMJOA treatment. Derived from a variety of tissues, MSCs exert therapeutic effects through diverse mechanisms, including chondrogenic differentiation; fibrocartilage regeneration; and trophic, immunomodulatory, and anti-inflammatory effects. Here, we provide an overview of the therapeutic roles of various tissue-specific MSCs in osteoarthritic TMJ or TMJ regenerative tissue engineering, with an additional focus on joint-resident stem cells and other cellular therapies, such as exosomes and adipose-derived stromal vascular fraction (SVF). Additionally, we summarized the updated pathogenesis of TMJOA to provide a better understanding of the pathological mechanisms of cellular therapies. Although limitations exist, MSC-centered therapies still provide novel, innovative approaches for TMJOA treatment.

## 1. Introduction

The temporomandibular joint (TMJ) is a synovial articulation that connects the mandibular condyle to the glenoid fossa of the temporal bone, and its inner space is divided into two compartments by a fibrocartilaginous TMJ disc [1]. It also has been considered as a bilateral diarthrodial joint according to its hinging and sliding movements and participation in essential life-support functions, such as chewing, swallowing, and speaking [2].

Osteoarthritis (OA) is a progressive degenerative condition that often causes deterioration of the cartilage and surrounding tissues, and TMJ is one of the most common sites affected by OA [3]. As a subtype of TMJ disorders (TMJDs), TMJOA is characterized by cartilage degeneration, disrupted subchondral bone remodeling, synovitis, and subsequent clinical symptoms, such as chronic orofacial pain, aberrant crepitations, restricted mandible motions, and functional disabilities [4, 5].

The diagnosis of TMJOA has mainly relied on evaluations of the aforementioned clinical features combined with radiographic assessments, and recent applications of cone-beam CT (CBCT) and MR imaging (MRI) further improved accuracy and sensitivity [6, 7]. Although the reported prevalence of TMJOA varies with the diverse diagnostic criteria, TMJOA still represents a growing health burden due to the limitations placed on patients' quality of life and the substantial socioeconomic costs [8].

Currently, the management of TMJOA is contingent on the severity. Conservative therapies, including medications, occlusal splints, intra-articular injections, arthrocentesis, or arthroscopy, are preferred for patients in early or medium stages, while radical curative methods, such as open joint surgeries, are only performed in severe cases, associated with high risks of complications [9–11]. However, due to the limited self-renewal capability of condylar structures, current treatments mainly focus on symptom alleviation and often fail to offer permanent recovery; thus, there is still a huge unmet clinical need for innovative approaches towards TMJOA [12].

As a large amount of literature has accumulated on the diverse functional roles played by stem cells, there is an emerging interest in the therapeutic effect of stem cells, mainly based on its immunoregulatory, anti-inflammatory, trophic activity, and multilineage differentiation potential [13–15]. Along with other cell-based therapies, increasing evidence has shed light on these regenerative medicines used in the field of TMJOA.

In this review, apart from a brief introduction about the unique construction and molecular composition of TMJ, we will discuss the latest research progress on the pathogenesis of TMJOA and summarize the diverse categories of stem cells and their pathological mechanism in TMJOA treatment, combined with other regenerative medicine, including exosomes, “minimally manipulated MSCs,” and platelet-rich plasma. Further, since MSC-centered cellular therapies are still in its infancy, we will also point out the limitations and controversies surrounding stem cell treatments, to provide an up-to-date review from a more objective perspective.

## 2. Pathogenesis of TMJOA

**Figure 1:**
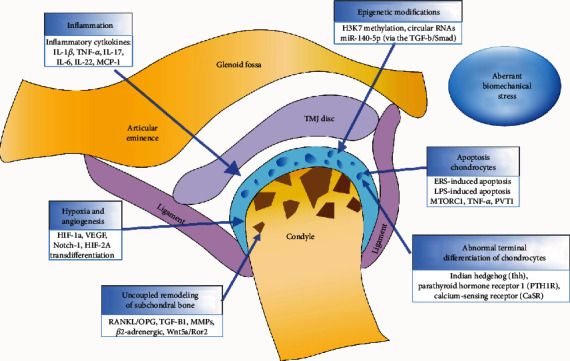
A schematic image of pathogenesis of TMJOA.

Due to the unique structure of the TMJ and its distinct composition of fibrocartilage, which predominantly contains type I collagen, the pathogenesis of TMJOA differs from the general OA that occurs in other joints, composed of hyaline cartilage to some extent [16]. The etiology of TMJOA is multifactorial [17], including risk factors from microtrauma, mechanical overload, malocclusion, bruxism, estrogen influence on systemic illness, and genetic variations [17–21]. Moreover, the pathogenesis of this disease is highly complicated and remains poorly understood, thus leading to current research focusing on underlying mechanisms (Figure 1). Therefore, we summarize several advances made in the last five years and add these to an outline from a previous review, especially regarding the aspects discussed below.

### 2.1. Inflammation

Although OA has long been considered a low-inflammatory and sterile disease [22], an increasing number of studies have suggested that inflammation is involved in the incidence and progression of TMJOA [23]. It was observed that a variety of inflammatory mediators and cytokines, such as tumor necrosis factor- (TNF-) *α*, interleukin- (IL-) 17, IL-6, IL-1*β*, and IL-22, are more abundant in the synovial fluid of TMJOA-afflicted patients [24–26], accompanied by infiltration of immune cells, predominantly macrophages and T lymphocytes [23].

It has been well established that inflammatory cytokines, such as IL-1*β*, released from stimulated immune cells play a critical role in cartilage destruction by increasing cartilage degradation and suppressing the synthesis of cartilage matrix in TMJOA, and this tissue catabolism aggravates inflammation in a vicious cycle [17]. Recent studies suggest that sustained inflammation deteriorates the ultrastructure and nanomechanical properties of collagen fibrils in TMJ discs, partially through changes in the microenvironment of the extracellular matrix [27, 28]. In addition, osteoarthritis pain is also in close correlation with TMJ inflammation, which may be initiated by the production of inflammatory mediators, such as prostaglandins and leukotrienes [24]. For instance, TNF-*α*, the earliest and most representative cytokine in TMJOA, can directly stimulate nociceptors [29] and is related to sensory neuron hyperexcitability [30]. Recently, it was found that sexual distinctions in inflammatory stimuli of synoviocytes may contribute to the gender difference in TMJOA. While increased proinflammatory factors and enhanced synovitis were observed in female rats, the synoviocytes from female rats also possess higher sensitivity to inflammatory stimuli in an estrogen-related manner, with more recruited mononuclear cells and correspondingly elevated monocyte chemoattractant protein- (MCP-) 1 levels [31].

### 2.2. Uncoupled Remodeling of Subchondral Bone

Changes in the osteochondral surface and subchondral bone are also a significant characteristic of TMJOA, whose clinical diagnosis can therefore be made on the basis of imaging findings [32]. While the remodeling process is always well-organized and maintains skeletal homeostasis under physiological conditions [33], imbalanced osteoblastic bone formation and osteoclast-mediated bone resorption can be observed in the osteoarthritic condyle. The loss of subchondral trabecular bone with decreased bone volume to tissue volume (BV/TV) and increased osteoclastic activity can be observed in experimental TMJOA animal models, such as unilateral anterior crossbite (UAC) rats, as confirmed in the literature [34–36]. A previous study reported that inhibited activity of local BMSCs decreased numbers of osteoblasts and osteoclasts at two weeks in UAC-treated cells [34]. However, it was observed that although the numbers of BMSCs and Ocn-positive osteoblasts declined, the osteogenic potential of BMSCs was stimulated, thereby strongly promoting the migration and differentiation of osteoclast precursors mediated by the Wnt5a/Ror2 signaling pathway, and finally resulting in an osteoclastic predominant, resorptive subchondral trabecular bone phenotype [37]. Moreover, researchers have also found that aberrant activation of transforming growth factor-*β* (TGF-*β*) in subchondral bone has participated in the initiation and progression of TMJOA [36, 38]. CED mice, an osteoblast-specific mutant TGF-*β*1 transgenic mouse model, displayed calcification degeneration of TMJ cartilage, loss of subchondral bone due to disrupted bone formation with high levels of active TGF-*β*, and inhibition of TGF-*β* receptors, which attenuated TMJOA development and restored bone mass.

Recently, studies indicated that sympathetic tone, which was shown to be associated with osteoarthritic pain [39], was also related to abnormal bone remodeling. It was found that *β*2-adrenergic signal transduction elicited subchondral bone loss and cartilage degradation by increasing RANKL secretion by MSCs in the condyle, while *β*-antagonist (propranolol) and conditional deletion of *β*2-adrenergic receptor (Adrb2) in nestin+ MSCs suppressed bone defects in the TMJ [40, 41]. Additionally, inflammatory mediators and immune responses also accelerate bone resorption via osteoclast activation [23]. Besides loss of trabecular bone, the disrupted balance of bone remodeling also leads to thickening, calcification, and stiffening of the osteochondral interface, as well as deformation of the condyle [36, 42]. Since the osteochondral interface and osteocytes are extremely sensitive to mechanical overload [23], the change in subchondral bone may exacerbate OA progression, which requires further confirmation [42].

### 2.3. Apoptosis and Anomalies of Chondrocytes

A major feature of TMJOA, the destruction of cartilage, was reported to be mainly attributed to apoptosis or other chondrocyte anomalies [43]. Several studies have indicated that through the activation of endoplasmic reticulum stress (ERS), some widely known pathogenic factors of OA, such as mechanical forces and hypoxia, can induce chondrocyte apoptosis and OA-like changes in the temporomandibular joint, and inhibition of the endoplasmic reticulum can reduce apoptosis and alleviate destruction of mandibular cartilage [44, 45]. A recent study has further explored the underlying mechanism of ERS pathway apoptosis (ERS-apoptosis) in TMJOA [46]. Experimental TMJOA models activated ERS and induced both ERS-apoptosis and protective autophagy in chondrocytes in the early stage, but the switch from autophagy to apoptosis, mediated by MTORC1, led to cartilage loss and progression of biomechanically induced TMJOA [47, 48]. In addition to ERS-apoptosis, inflammation is involved in the pathogenesis of TMJOA; a recent study also found that inflammatory synoviocytes in TMJ could facilitate lipopolysaccharide- (LPS-) induced apoptosis of chondrocytes by increasing TNF-*α*, which is regulated by long noncoding RNA plasmacytoma variant translocation 1 (PVT1) [49, 50].

Moreover, abnormal terminal differentiation of chondrocytes also contributes to cartilage degeneration in TMJOA, while expression levels of Col-X, MMP13, and ALP, the markers that represent the hypertrophic stage, are often elevated in OA cartilage [51–53]. Studies have revealed that accelerated terminal differentiation in chondrocytes, which leads to cartilage degeneration, is one of the typical characteristics in TMJOA animal models, and the underlying molecular mechanism is related to Indian hedgehog (Ihh), parathyroid hormone receptor 1 (PTH1R) signaling, and calcium-sensing receptor (CaSR), induced by aberrant biomechanical stress [54–56].

### 2.4. Hypoxia and Angiogenesis

Recent studies have also focused on the complicated role played by hypoxia and angiogenesis in the onset and development of TMJOA. While some studies pointed out that HIF1*α* may maintain cartilage homeostasis in a protective way, other researchers have indicated that the HIF1-VEGF-Notch pathway accelerates TMJOA progression [57, 58]. In experimental TMJOA rats, expression levels of HIF1*α*, VEGF, and Notch-1 in hypoxic chondrocytes were significantly higher, together with pathological angiogenesis at the osteochondral junction [58–60]. Inhibition of HIF1*α* or Notch pathways can partially delay the progression of OA [61].

Another hypoxia inducible factor, HIF2*α*, also participates in cartilage destruction via multiple mechanisms. As osteoarthritic changes can be triggered by an imbalance between catabolic and anabolic factors, HIF2*α* directly induces the upregulation of matrix metalloproteinases (MMPs), aggrecanase-1 (ADAMTS4), nitric oxide synthase-2 (NOS2), and other catabolic factors, thereby exacerbating OA progression [62–64]. Notably, excessive TMJ loading and estrogen have also been proven to initiate or aggravate TMJOA in a HIF2*α*-related manner, suggesting intricate interactions among these pathological mechanisms [62, 65, 66]. Recently, a study revealed the existence of fully formed vasculature within both healthy and pathological TMJ condylar cartilage in higher order species, such as miniature pigs and humans, which participates in driving chondrocyte to osteoblast transdifferentiation and the pathogenesis of TMJOA. Therefore, antiangiogenesis therapies that inhibit cartilage-to-bone transformation may be involved in the management of TMJOA in the future [67].

### 2.5. Epigenetic Modification

Recently, as accumulating studies have focused on epigenetics, its relationship with TMJOA has aroused attention, generating a new perspective for the pathogenesis of degenerative TMJOA. Genome-scale DNA methylation profiles in condylar cartilage have revealed dynamic DNA methylation patterns with differentially expressed genes in the early, intermediate, and late phases of TMJOA [68]. In addition, a study demonstrated that regulation of histone H3 lysine 9 (H3K9) methylation participates in TMJ cartilage homeostasis in terms of cell growth, apoptosis, and gene expression, and has potentially provided a novel curative option for TMJOA [69]. Moreover, a recent study has also pointed out that circular RNAs, a kind of noncoding RNA widely present in eukaryotic cells, which have been shown to play a role in limb and facet joint osteoarthritis, are also involved in the progression of TMJOA by elevating TNF-*α* secretion by the synovium via the ceRNA mechanism [70–74]. Moreover, in 2019, Li et al. found that miR-140-5p, which also belongs to the noncoding RNAs and was previously demonstrated to participate in different stages of OA development, was capable of regulating TMJOA pathogenesis via the TGF-*β*/Smad signaling pathway, through mediating mandibular condylar cartilage homeostasis [75].

## 3. Mesenchymal Stem Cell (MSC) Application for TMJOA

Mesenchymal stem cells (MSCs) are multipotent progenitor cells with the capability of self-renewal and differentiation into multiple lineages. As MSCs are highly heterogeneous populations, finding a single unambiguous phenotypic marker for MSCs is still an unsettled issue; therefore, identification is based on a panel of both positive and negative markers [15]. As recommended by the International Society of Cellular Therapy, MSCs should be identified as plastic-adherent cells, with expressions of CD73, CD90, and CD105 and the absence of hematopoietic markers and HLA class II molecules, and possess tripotent differentiation into chondrogenic, osteogenic, and adipogenic phenotypes [76, 77]. In the past few years, numerous preclinical and clinical studies have been conducted on the utility of MSCs in osteoarthritis of other joints, such as the knee meniscus, demonstrating the therapeutic effects of MSCs on cartilage regeneration, symptom alleviation, and pain management [10, 78–81]. The regenerative characteristics of MSCs and these optimistic results have catapulted them to the forefront of TMJOA cellular treatment in recent years.

### 3.1. Bone Marrow Mesenchymal Stem Cells (BMMSCs)

Currently, bone marrow is the most common MSC source in clinical practice and has been widely studied in the field of cartilage repair, either alone or with scaffolds. Although there are limitations, such as donor site morbidity, bone marrow mesenchymal stem cells (BMMSCs) are superior in cell proliferation and chondrogenic differentiation ability [82]. Notably, immune escape is a significant feature of BMMSCs, which allows for clinical applications of xenogenic MSCs, without resulting in an obvious inflammatory reaction [83–85]. Several studies have indicated that an exogenous supply of BMMSCs exerts therapeutic effects on TMJOA via multiple mechanisms [48, 83, 86]. It has been reported that continual BMMSC injections rescued cartilage degradation and abnormal subchondral bone remodeling, leading to improved BV/TV, Tb. Sp, and cartilage thickness, together with decreased loss of glycosaminoglycans [83]. Researchers further determined that the reparative effect of BMMSCs on cartilage mainly relies on promoting cartilage matrix production and activating protective scavenging activity, resulting in a reduction in chondrocyte apoptosis in the replenished matrix [48]. Moreover, BMMSCs display immunomodulatory effects by secreting anti-inflammatory factors [87]. Upregulated levels of TNF-*α* and IL-1*β* in mice with osteoarthritic TMJ returned to control levels after the administration of BMMSC injections [83]. In addition, studies have suggested that the direct incorporation and differentiation of chondrocytes may also participate in the therapeutic process [88], and it was observed that chondrogenically differentiated MSCs were more effective in delaying the progression of TMJOA *in vitro*, especially during the early period [86].

Apart from injections, tissue engineering provides a final-stage solution for severe TMJOA; BMMSCs combined with tissue engineering techniques exhibited promising effects on TMJOA treatment. A previous study showed that a BMMSC-containing scaffold with a periosteal or synovial flap led to fibrocartilage tissue repair 12 months postsurgery [89]. Combined with a microprecise spatiotemporal delivery system for growth factors embedded in 3D printed TMJ scaffolds, bone marrow-derived and synovium-derived MSCs can be induced into regionally controlled differentiation, resulting in the formation of multiphase fibrocartilaginous tissues [90]. Similarly, it was observed that BMMSCs seeded in 3D-printed scaffolds with growth factors, such as transforming growth factor- (TGF-) *β*3 and connective tissue growth factor (CTGF), resulted in a heterogeneous fibrocartilaginous matrix, further proving its potential for TMJ tissue engineering and TMJOA treatment [91].

### 3.2. Adipose Stem Cells (ASCs)

Adipose tissue is also a highly attractive source of mesenchymal stem cells [92]. While the immunophenotypes of BMMSCs and ASCs are more than 90% identical, compared to BMMSCs, adipose-derived stem cells (ASCs) that present multilineage differentiation are easily obtainable, owing to the ubiquitous adipose tissue in the body and ease of extraction and isolation [93–95]. Besides, the strengths of ASCs also include their abundance in adipose tissue, manipulability, rapid growing ability, and less interference by age than BMMSCs [96–98]. Notably, ASCs are capable of replicating the extracellular matrix environment of implanting sites with different types of collagen and undergoing hypoxic conditions, which makes implantation possible in the lowly vascularized TMJ structure [99]. In recent years, ASCs have been shown to represent a promising therapeutic strategy for osteoarthritis [100]. Many clinical trials or case reports demonstrated that intra-articular injections of ASCs into patients with knee osteoarthritis led to safe and satisfactory results involving functional improvement, pain relief, and delayed progression of OA, without severe adverse effects reported yet [78, 101–103].

In 2010, an *in vitro* study showed that adipose stem cells cultured in chondrogenic conditions combined with polylactide (PLA) discs can be used in TMJ disc engineering, creating constructs that can replace degenerated TMJ discs [99]. The authors further conducted a similar *in vivo* study using ASCs cultured in PLA discs in TMJs of rabbits. These results proved that the use of ASCs, especially predifferentiated with TGF-*β*1 in TMJ engineering, promoted fibrocartilaginous TMJ disc-like tissue formation [104]. More recently, a study obtained positive outcomes after autologous ASC infiltration in the TMJ superior compartment of patients suffering from temporomandibular disorders [105]. They found that ASC injections with arthrocentesis led to the alleviation of symptoms and restoration of articular anatomical integrity, as demonstrated by MRI findings. However, since the study focused on TMJ internal derangement (ID) and is associated with a relatively high risk of bias, further investigations are required regarding the applications of ASCs for TMJOA treatment.

### 3.3. Umbilical Cord Mesenchymal Stem Cells (UC-MSCs)

Umbilical cord mesenchymal stem cells (UC-MSCs), which can be obtained from various umbilical cord compartments, such as Wharton's jelly (WJ), perivascular tissue (PVT), and endovascular compartment (UCB), through a painless and safe procedure, are also a promising stem cell resource in regenerative medicine [106–108]. In addition, compared to other MSCs, UC-MSCs are a relatively young cell type with great proliferation and differentiation capability, and have no ethical concerns or tumorigenesis possibility, unlike embryonic stem cells (ESCs) or induced pluripotent stem cells (iPSCs) [109–112].

Previous studies have proved that instead of generating hyaline cartilage, the strong potential for fibrocartilage production by UC-MSCs has turned them into a desirable option for TMJ tissue engineering [111, 113]. A study by Bailey et al. suggested that in comparison with TMJ condylar chondrocytes, UC-MSC-seeded PLA constructs displayed higher levels of biosynthesis and cellularity [114]. An *in vitro* study by Wang et al. using 3D scaffolds also revealed that UC-MSCs outperformed BMMSCs in terms of fibrocartilage regeneration, with greater amounts of type I collagen and aggrecan produced [113]. They also found that a higher initial seeding density of UC-MSCs led to enhanced mechanical integrity and biosynthesis of newly synthesized fibrocartilage [115]. Recently, a study by Kim et al. demonstrated that intra-articular UC-MSC injections exert therapeutic potential in a rabbit model of TMJOA [116]. With upregulated expression of growth factors, extracellular matrix markers, and anti-inflammatory cytokines, transplanted UC-MSCs not only ameliorated degeneration of cartilage and subchondral bones, but also exerted pronounced anti-inflammatory effects, analogous to dexamethasone (DEX). Additionally, the restorative effects can last for a minimum of four weeks, without local or general complications. Some osteoarthritic animal models also confirmed this curative effect by UC-MSCs, showing improved structures, suppressed inflammation, and relatively promoted clinical signs [117, 118].

### 3.4. Tooth-Derived Mesenchymal Stem Cells

A great variety of tooth-derived stem cells, including periodontal ligament stem cells (PDLSCs), dental pulp stem cells (DPSCs), dental follicle progenitor cells (DFPCs), and dental pulp stem cells from deciduous teeth (SHED), have been proposed as promising candidates in the field of regenerative medicine [119–121]. In addition to their proliferation and self-renewal capacities similar to BMMSCs, tooth-derived stem cells, such as PDLSCs, SHEDs, and DPSCs, are also capable of differentiating into chondrocytes and osteoblasts, and therefore adequate for tissue repair outside of tooth structures [122–125]. Moreover, compared to BMMSCs, tooth-derived stem cells may display a high affinity for regenerating craniofacial tissues, based on the same embryological origins they share and similar gene expression patterns [126, 127].

Although previous studies have focused on the potential of dental stem cells in articular cartilage repair [125, 128, 129], Bousnaki et al. first revealed applications of dental pulp stem cells (DPSCs) in TMJ tissue engineering [130]. DPSCs exhibited enhanced fibro-/chondrogenic differentiation and abundant fibrocartilaginous tissue formation when seeded into chitosan/alginate (Ch/Alg) scaffolds with an interconnected porous structure. Compared to cell-free scaffolds, constructs with DPSCs obtained markedly promoted storage modulus and elastic response, similar to natural TMJ discs.

Notably, some studies revealed that instead of directly differentiating into chondrocytes, dental stem cells participated in the TMJ regeneration process via a paracrine mechanism [131, 132]. The trophic roles of MSCs have been extensively investigated [133–136]. A study by Zhang et al. demonstrated that periodontal ligament stem cells (PDLSCs) facilitate fibrocartilage regeneration in TMJ disorders by stimulating proliferation and extracellular matrix formation of TMJ-derived chondrocytes [131]. Similarly, it was found that factors secreted by stem cells from human exfoliated deciduous teeth (SHED), a kind of MSC residing in the perivascular niche of dental pulps, exhibited therapeutic effects for TMJOA. Intravenous administration of serum-free conditioned media from SHED (SHED-CM) inhibited cartilage devastation and temporal muscle inflammation, thus regenerating condylar cartilage and subchondral bone without adverse side effects [132]. These results together shed light on the possible applications of tooth-derived stem cells in TMJ osteoarthritis and regeneration therapies.

### 3.5. Joint-Derived Mesenchymal Stem Cells

Besides the aforementioned tissues, the joint itself also harbors a rich mesenchymal stem cell reservoir, such as MSCs in synovial fluid [137], synovium [138], or fibrocartilage [139, 140], which have been found to participate in joint homeostasis and self-repair processes [141, 142]. Synovial fluid mesenchymal stem cells (SF-MSCs) and serum-deprived mesenchymal stem cells (SD-MSCs) have gained much interest in cell-based therapies for degenerative joint disease as these cells boast superior chondrogenic capacity [143], decreased hypertrophic potential [144], and easier harvest procedures [145] compared to BMMSCs. Previous studies on the effects of joint-derived MSCs on meniscal regeneration or osteoarthritic large joints have yielded encouraging outcomes [146–148]; TMJ-synovial stem cells from TMD patients also exhibited similar potential in TMJ disc regeneration. An *in vivo* study showed that TMJ-synovial stem cells underwent chondrogenic differentiation and produced cartilage ECM when seeded in fibrin/chitosan scaffolds, demonstrating a regenerative ability for perforated TMJ discs, often seen in the late stage of TMJOA [149].

Aside from applications with extracted and subsequently implanted MSCs, manipulation of joint-resident stem cells may also serve as a novel therapeutic target for TMJOA. It is generally acknowledged that joint-resident MSCs could be mobilized by multiple recruiting signals and exert reparative effects on destroyed cartilage through chondrogenic differentiation, ECM reconstruction, and immunomodulation [142]. This could be partially confirmed by increasing amounts of MSCs in osteoarthritic synovium or synovial fluids [150]. While joint irrigation with arthroscopy has been a classical method for osteoarthritis, the side effect of lavage-depleting joint-resident SF-MSCs, may lead to undesirable results that go against traditional experience [151]. Accordingly, Baboolal et al. developed a novel arthroscopic technique aimed at mobilizing resident SF-MSCs to promote the endogenous repair potential of joints [152].

However, this reparative capacity of joint-resident MSCs could be inhibited by one of the key factors in the pathogenesis of TMJOA and inflammation. It was found that proinflammatory cytokines, such as IL-1*β*, impede the chondrogenic potential of SF-MSCs or SD-MSCs in osteoarthritic TMJ, and make these MSCs in osteoarthritic joints further secrete proinflammatory cytokines such as IL-6 or IL-8 [153–155]. Therefore, medications targeting inflammatory pathways in MSCs, such as histone deacetylase inhibitors LBH589, SAHA, and MC1568, may provide new insights into TMJOA treatment by attenuating MSC-secreted cytokines [156, 157]. Interestingly, Embree et al. identified a type of resident fibrocartilage stem cell (FCSC) in the superficial zone niche of the TMJ condyle. The depletion of these cells by overactive Wnt signals would disturb the homeostasis of TMJ fibrocartilage and result in its degeneration. Based on this newly proposed pathological mechanism, exploiting resident FCSCs with exogenous canonical Wnt inhibitor sclerostin (SOST) for condylar cartilage repair may present a novel cell-based therapy [140, 158, 159].

## 4. Other Cellular-Based Therapies for TMJOA

### 4.1. Exosomes

As previously discussed, an increasing amount of literature has indicated that, rather than direct chondrogenic differentiation, the therapeutic efficacy of MSCs is predominantly attributed to paracrine secretions, where exosomes play a major role. Exosomes are nanosized (30-150 nm) extracellular vesicles (EVs), released through the fusion of multivesicular bodies with the plasma membranes from various cell types [160]. With their complex cargos and bilipid membrane structures, exosomes primarily function as intercellular communication vehicles, transferring bioactive lipids, nucleic acids (mRNAs and microRNAs), and proteins between cells under both physiological and pathological conditions [161, 162].

To date, exosomes have been isolated from different types of stem cells, including ASCs [163], BMMSCs [164], UC-MSCs [165], ESCs [166], and iPSCs [167]. These stem cell-derived exosomes exert therapeutic effects against various diseases, such as myocardial ischemia/reperfusion injury [168] and renal injury [165], and promote cartilage and bone regeneration [167, 169]. In recent years, numerous preclinical studies on the application of MSC exosomes for cartilage lesions and knee OA have shown promising outcomes, demonstrating that with different cargos, exosome injections led to symptom alleviation, inflammatory suppression, and cartilage regeneration [10, 162, 166, 170]. Thus, exosomes have also catapulted to the forefront of TMJOA cellular therapies, although the research is relatively limited.

In 2019, a study by Zhang et al. first revealed the therapeutic role of human MSC exosomes in TMJOA [13]. Through a well-orchestrated mechanism that targets several critical features of TMJOA pathology, MSC exosomes alleviate osteoarthritic TMJ deterioration and promote repair and regeneration. These protective effects of MSC exosomes can be partially attributed to adenosine receptor-dependent AKT, ERK, and AMPK signaling pathways. Additionally, the MSC exosomes also exhibited immunomodulatory activity during the osteochondral process, as they can elicit infiltration of regenerative and anti-inflammatory M2 macrophages over M1 macrophages, whose polarization in osteoarthritic tissue inhibits chondrogenic differentiation of MSCs [171–173]. In addition, the complex cargos of MSC exosomes may serve as major curative agents for TMJOA. Luo et al. recently found that exosomes derived from human exfoliated deciduous teeth (SHEDs) exert anti-inflammatory effects in TMJOA [174], which rely on the exosomal cargo, miR-100. miR-100 in SHED-exosomes suppresses the expression of proinflammatory cytokines and catabolic enzymes, such as IL-6; IL-8; and MMP1, 3, 9, and 13, via repression of mammalian target of rapamycin (mTOR) pathways.

### 4.2. Minimally Manipulated MSC (BMAC and SVF)

Although in recent years, MSC-based therapy has rapidly progressed in the field of OA and cartilage regeneration, with considerable optimistic outcomes from animal and human studies, the clinical applications of MSCs are still under strict restrictions by the Food and Drug Administration (FDA), particularly with regard to cell expansion [175]. This regulatory limitation has incentivized clinicians to develop alternative cell-based methods with MSCs, the so-called minimally manipulated MSCs [176], which means that cells are directly manipulated near the operating room prior to implantation, instead of expansion before. Bone marrow aspirate concentrate (BMAC) and adipose-derived stromal vascular fraction (SVF) are two major modalities of minimally manipulated MSCs.

Obtained through bone marrow needle aspirates and subsequent centrifugation [177], BMAC has complex cellular elements, including BMMSCs and other components such as bone marrow-derived platelets [176]. While many researchers have reported positive results using BMAC in knee osteoarthritis patients, such as improvement in pain and function [178–180], a randomized controlled trial with 12-month follow-up by Shapiro et al. indicated that no significant differences existed between BMAC- and saline-treated patients [181, 182]. Moreover, as no clinical trials involving BMACs have been conducted, there is also a vacancy in BMAC application in osteoarthritis TMJ or fibrocartilage regeneration, requiring further studies in this regard. Conversely, clinical applications of SVF have produced more stable and satisfactory outcomes in OA or cartilage regeneration, relative to BMAC. SVF, a potent regenerative solution containing ASCs, is a component of lipoaspirate, the collection of which involves multiple steps, such as liposuction, digestion of the extracellular matrix (ECM), and centrifugation [84]. An alternative SVF isolation method is nanofragmented adipose tissue (nanofat), with retained ECM to maintain the intact microenvironment of stem cells [183].

Several studies have indicated positive clinical outcomes of SVF administration in knee OA cases, with improvements in articular function, clinical symptoms, MRI results, and even histological findings from knee biopsies [184–187]. However, a recent systematic review pointed out that a lack of high-quality and well-designed clinical studies weakened the credibility of encouraging results after BMAC or SVF applications [176]. Additionally, the limited number of research participants and concomitant use of other curative agents, such as dexamethasone, hyaluronic acid (HA), and platelet-rich plasma (PRP), also undermined the distinct identification of their effects [84]. More recently, studies have also started to focus on the potential effect of SVF on TMJ disorders. A study evaluated the efficacy of intra-articular nanofat injection for patients with TMD and found a remarkable improvement in all four parameters (pain level, maximum mouth opening (MMO), joint clicking, and deviation), without significant side effects or complications [188]. In addition, another *in vitro* study published in 2020 reported that SVF, which contained 32% ASCs via their modified isolation method, downregulated the expression of OA-related inflammatory cytokines, such as PGE2 and CXCL8/IL-8, in osteoarthritic TMJ synoviocytes [189]. The PGE8 decrease was more prominent in SVF than in ASCs, suggesting that other components of SVF participated in the anti-inflammatory effect. Overall, although further *in vivo* and preclinical studies are needed, these studies revealed that SVF may be a feasible cellular therapy for TMJOA in the future.

## 5. Limitations of Cell-Based Therapies for TMJOA

In recent years, despite increasing studies that have shed light on the potential capability of cell-based therapies for TMJOA, unfortunately, many problems still impede its application in clinical settings. One of the major obstacles in MSC application is the absence of a specific and clear understanding of the therapeutic mechanisms, due to the complexity of MSC metabolism and difficulty of cell fate mapping. As previous studies primarily anchored their hope on the multilineage differentiation ability of MSCs [86], some researchers have pointed out that the retention rate of transplanted MSCs in the OA joint was relatively low, suggesting that the therapeutic role relies more on alternative mechanisms, such as a paracrine effect [15].

Another major problem is that the hostile environment of osteoarthritic joints may undermine the functions of MSCs, either joint-resident or transplanted, such as their viability, chondrogenic ability, and immunomodulatory effects [141, 142]. Increasing evidence suggests that MSCs may upregulate inflammation under certain OA conditions, and may even differentiate into a proinflammatory phenotype or become a new source of inflammatory cytokines, as discussed [153, 190, 191]. Some studies have attempted to solve this problem by optimizing MSCs with signaling pathway inhibition, such as STAT3, which yields satisfactory outcomes [192]. However, further research regarding the underlying mechanism is still required. Many basic questions about cellular therapies are still calling for definite answers, such as the optimal cell source, cell dose, injection times, or intervals. Additionally, the lack of a consistent approach in trial designs presents a hurdle to the adoption of these treatments. As MSC exosomes represent a more accessible, “off-the-shelf,” and cell-free MSC therapy, significant challenges and considerations in terms of manufacture, safety, long-term durability, and regulation still remain, which should be thoroughly discussed before its translation to clinical trials [193].

## 6. Conclusions

Due to the complicated pathogenesis of TMJOA and poor self-healing capability of a condylar structure, current management for this degenerative disease, both conservative and surgical, are mainly symptomatic therapies. Unmet clinical needs for effective, long-term, disease-modifying strategies to regenerate the osteoarthritic TMJ structure still exist, and therefore lead to the increasing interest in cellular-based therapies, namely mesenchymal stem cells (MSCs) and their related derivatives, such as exosomes and minimally manipulated MSCs. Applications of exogenous MSCs from abundant sources alone or in conjugation with tissue engineering have exhibited promising results in preclinical studies involving TMJOA, revealing rescued cartilage degradation, pronounced fibrocartilaginous tissue or matrix formation, and decreased inflammatory levels (Table 1). Although the reparative effects were mainly ascribed to chondrogenic differentiation of MSCs, current studies tend to emphasize the paracrine mechanisms of MSCs. Furthermore, interventions that harnessed endogenous joint-derived MSCs or exosomes also exhibited remarkable potential for TMJOA treatments.

Although many hurdles regarding the principles and specific usages of MSCs still remain, and nearly all the aforementioned studies rest on the preclinical stage, current evidence for these cellular-based therapies reveals the feasibility of revolutionizing traditional therapeutic options for TMJOA. However, further *in vivo* studies are required to overcome existing problems, in order to move these methods from bench to bedside, both efficiently and safely.

## Figures and Tables

**Table 1:** An overview of various tissue-specific MSCs utilized in osteoarthritic TMJ or TMJ regenerative tissue engineering.

Cell	Interventions			Improvement			Refs.
	Operative ways	Animal	Dosage	Cartilage	Subchondral bone	Inflammation	
Bone marrow mesenchymal stem cells (BMSCs)	Local injections of 2∗10^3^ exogenous BMSCs	Mouse	Weekly, 3 weeks after OA induction; continuing for 4-, 8- and 12-weeks.	Yes	Yes	Yes	[48, 83]
	Local injection of 2∗10^5^ autologous BMSC	Rabbit	One time, 4 weeks after OA induction	Yes	Yes	N/A	[86]
	2∗10^6^ cells/ml BMSCs with 3D-printed scaffolds	Rabbit	One time, cultured for 6 weeks with 1 : 1 mixture of fibrogenic and chondrogenic supplements	Yes	N/A	N/A	[90, 91]

Adipose stem cells (ASCs)	1∗10^5^ ASCs with TMJ disc-shaped bilayer biodegradable PLA disc	In vitro	Cultured for a maximum of 6 weeks	Yes	N/A	N/A	[99, 104]
	4∗10^3^ ASCs with bilayer PLA discs	Rabbit	One time, follow-up periods of 6 (*n* = 5) and 12 months (*n* = 5)	Yes	Yes	N/A	[104]
	Local injections of 1 cc autologous ASCs by arthrocentesis	Human	One time, 6 month follow-up	Yes	Yes	Yes	[105]

Umbilical cord Mesenchymal stem cells (UC-MSCs)	3.4∗10^6^ UC-MSCs with PGA scaffolds	In vitro	Seeded in the scaffolds for 6 days and cultured for 4 weeks	Yes	N/A	N/A	[114]
	Intra-articular injections of 2∗10^4^ ~ 2∗10^5^ UC-MSCs	Rabbit	One time, injections at 4 weeks after OA induction; sacrificed at 8 weeks after OA induction	Yes	Yes	Yes	[116]

Tooth-derived mesenchymal stem cells (including PDLSCs, DPSCs, DFPCs, and SHEDs)	2∗10^6^ DPSCs seeded in chitosan/alginate scaffolds with an interconnected porous structure	In vitro	DPSCs were cultured for 21days before seeding; the DPSCs/scaffold constructs were incubated for up to 8 weeks	Yes	N/A	N/A	[130]
	1∗10^5^ PDLSCs	In vitro	Cocultured with 1∗10^5^ fibrochondrocytes for 3 weeks	Yes	N/A	N/A	[131]
	Tail vein injection of conditioned media from SHED	Rat	Daily, from day 6 to day 10	Yes	Yes	Yes	[132]

Joint-derived mesenchymal stem cells	2∗10^6^ MSCs from the TMJ-synovial membrane with fibrin/chitosan scaffolds	Rat	One time, 4 weeks of subcutaneous implantation	Yes	N/A	N/A	[149]
	Intra-articular treatment with the Wnt inhibitor sclerostin (SOST)	Rabbit	Weekly, from week 1 to week 7	Yes	N/A	N/A	[140]

Exosomes	Intra-articular injections of 100 *μ*g exosomes	Rat	Weekly, 2 weeks after OA induction, continuing for 2, 4, or 8 weeks	Yes	Yes	Yes	[13]

## Data Availability

No data were used to support this study.

## References

[1] Aryaei A., Vapniarsky N., Hu J. C., Athanasiou K. A. (2016). Recent tissue engineering advances for the treatment of temporomandibular joint disorders. *Current Osteoporosis Reports*.

[2] Roberts W. E., Stocum D. L. (2018). Part II. Temporomandibular joint (TMJ)-regeneration, degeneration, and adaptation. *Current Osteoporosis Reports*.

[3] Hunter D. J., Bierma-Zeinstra S. (2019). Osteoarthritis. *The Lancet*.

[4] Zarb G. A., Carlsson G. E. (1999). Temporomandibular disorders: osteoarthritis. *Journal of Orofacial Pain*.

[5] Chung P.-Y., Lin M.-T., Chang H.-P. (2019). Effectiveness of platelet-rich plasma injection in patients with temporomandibular joint osteoarthritis: a systematic review and meta-analysis of randomized controlled trials. *Oral surgery, Oral Medicine, Oral Pathology and Oral Radiology*.

[6] Arayasantiparb R., Mitrirattanakul S., Kunasarapun P., Chutimataewin H., Netnoparat P., Sae-Heng W. (2020). Association of radiographic and clinical findings in patients with temporomandibular joints osseous alteration. *Clinical Oral Investigations*.

[7] Suenaga S., Nagayama K., Nagasawa T., Indo H., Majima H. J. (2016). The usefulness of diagnostic imaging for the assessment of pain symptoms in temporomandibular disorders. *Japanese Dental Science Review*.

[8] de Souza R. F., Lovato da Silva C. H., Nasser M., Fedorowicz Z. (2012). Interventions for the management of temporomandibular joint osteoarthritis. *The Cochrane Database of Systematic Reviews*.

[9] Ouanounou A., Goldberg M., Haas D. A. (2017). Pharmacotherapy in temporomandibular disorders: a review. *Journal (Canadian Dental Association)*.

[10] Zhang R., Ma J., Han J., Zhang W., Ma J. (2019). Mesenchymal stem cell related therapies for cartilage lesions and osteoarthritis. *American Journal of Translational Research*.

[11] Haigler M. C., Abdulrehman E., Siddappa S., Kishore R., Padilla M., Enciso R. (2018). Use of platelet-rich plasma, platelet-rich growth factor with arthrocentesis or arthroscopy to treat temporomandibular joint osteoarthritis: systematic review with meta-analyses. *Journal of the American Dental Association*.

[12] Murphy M. K., MacBarb R. F., Wong M. E., Athanasiou K. A. (2013). Temporomandibular disorders: a review of etiology, clinical management, and tissue engineering strategies. *The International Journal of Oral & Maxillofacial Implants*.

[13] Zhang S., Teo K. Y. W., Chuah S. J., Lai R. C., Lim S. K., Toh W. S. (2019). MSC exosomes alleviate temporomandibular joint osteoarthritis by attenuating inflammation and restoring matrix homeostasis. *Biomaterials*.

[14] Zhang S., Yap A. U. J., Toh W. S. (2015). Stem cells for temporomandibular joint repair and regeneration. *Stem Cell Reviews and Reports*.

[15] Barry F. (2019). MSC therapy for osteoarthritis: an unfinished story. *Journal of Orthopaedic Research : Official Publication of the Orthopaedic Research Society*.

[16] van Bellinghen X., Idoux-Gillet Y., Pugliano M. (2018). Temporomandibular joint regenerative medicine. *International Journal of Molecular Sciences*.

[17] Tanaka E., Detamore M. S., Mercuri L. G. (2008). Degenerative disorders of the temporomandibular joint: etiology, diagnosis, and treatment. *Journal of Dental Research*.

[18] Dias G. M., Bonato L. L., Guimarães J. P. (2015). A study of the association between sleep bruxism, low quality of sleep, and degenerative changes of the temporomandibular joint. *The Journal of Craniofacial Surgery*.

[19] Barrera-Mora J. M., Escalona E. E., Labruzzi C. A. (2012). The relationship between malocclusion, benign joint hypermobility syndrome, condylar position and TMD symptoms. *Cranio*.

[20] Ikeda Y., Yonemitsu I., Takei M., Shibata S., Ono T. (2014). Mechanical loading leads to osteoarthritis-like changes in the hypofunctional temporomandibular joint in rats. *Archives of Oral Biology*.

[21] Hong S. W., Lee J. K., Kang J.-H. (2019). Skeletal maturation and predicted adult height in adolescents with temporomandibular joint osteoarthritis. *Journal of Oral Rehabilitation*.

[22] Savage J., McCullough M., Dimitroulis G. (2015). Microbiological investigation of the mandibular condyle in patients with advanced osteoarthritis of the temporomandibular joint. *International Journal of Oral & Maxillofacial Surgery*.

[23] Alvarez C., Monasterio G., Cavalla F. (2019). Osteoimmunology of oral and maxillofacial diseases: translational applications based on biological mechanisms. *Frontiers in Immunology*.

[24] Ibi M. (2019). Inflammation and temporomandibular joint derangement. *Biological & Pharmaceutical Bulletin*.

[25] Sorenson A., Hresko K., Butcher S. (2018). Expression of interleukin-1 and temporomandibular disorder: contemporary review of the literature. *Cranio*.

[26] Monasterio G., Castillo F., Rojas L. (2018). Th1/Th17/Th22 immune response and their association with joint pain, imagenological bone loss, RANKL expression and osteoclast activity in temporomandibular joint osteoarthritis: a preliminary report. *Journal of Oral Rehabilitation*.

[27] Wang X. D., Kou X. X., Mao J. J., Gan Y. H., Zhou Y. H. (2012). Sustained inflammation induces degeneration of the temporomandibular joint. *Journal of Dental Research*.

[28] Cui S. J., Fu Y., Liu Y. (2019). Chronic inflammation deteriorates structure and function of collagen fibril in rat temporomandibular joint disc. *International Journal of Oral Science*.

[29] Durham Z. L., Hawkins J. L., Durham P. L. (2017). Tumor necrosis factor-alpha stimulates cytokine expression and transient sensitization of trigeminal nociceptive neurons. *Archives of Oral Biology*.

[30] Sperry M. M., Kartha S., Winkelstein B. A., Granquist E. J. (2019). Experimental methods to inform diagnostic approaches for painful TMJ osteoarthritis. *Journal of Dental Research*.

[31] Xue X. T., Zhang T., Cui S. J. (2018). Sexual dimorphism of estrogen-sensitized synoviocytes contributes to gender difference in temporomandibular joint osteoarthritis. *Oral Diseases*.

[32] Larheim T. A., Abrahamsson A. K., Kristensen M., Arvidsson L. Z. (2015). Temporomandibular joint diagnostics using CBCT. *Dento Maxillo Facial Radiology*.

[33] Lerner U. H., Kindstedt E., Lundberg P. (2019). The critical interplay between bone resorbing and bone forming cells. *Journal of Clinical Periodontology*.

[34] Yang T., Zhang J., Cao Y. (2014). Decreased bone marrow stromal cells activity involves in unilateral anterior crossbite-induced early subchondral bone loss of temporomandibular joints. *Archives of Oral Biology*.

[35] Jiao K., Niu L. N., Wang M. Q. (2011). Subchondral bone loss following orthodontically induced cartilage degradation in the mandibular condyles of rats. *Bone*.

[36] Zheng L., Pi C., Zhang J. (2018). Aberrant activation of latent transforming growth factor-*β* initiates the onset of temporomandibular joint osteoarthritis. *Bone Research*.

[37] Yang T., Zhang J., Cao Y. (2015). Wnt5a/Ror2 mediates temporomandibular joint subchondral bone remodeling. *Journal of Dental Research*.

[38] Jiao K., Zhang M., Niu L. (2014). Overexpressed TGF-*β* in subchondral bone leads to mandibular condyle degradation. *Journal of Dental Research*.

[39] Aso K., Shahtaheri S. M., Hill R., Wilson D., McWilliams D. F., Walsh D. A. (2019). Associations of symptomatic knee osteoarthritis with histopathologic features in subchondral bone. *Arthritis & Rhematology*.

[40] Sun J.-L., Yan J. F., Li J. (2020). Conditional deletion of Adrb2 in mesenchymal stem cells attenuates osteoarthritis-like defects in temporomandibular joint. *Bone*.

[41] Jiao K., Niu L. N., Li Q. H. (2015). *β*2-Adrenergic signal transduction plays a detrimental role in subchondral bone loss of temporomandibular joint in osteoarthritis. *Scientific Reports*.

[42] Zhang J., Liao L., Zhu J. (2018). Osteochondral interface stiffening in mandibular condylar osteoarthritis. *Journal of Dental Research*.

[43] Charlier E., Relic B., Deroyer C. (2016). Insights on molecular mechanisms of chondrocytes death in osteoarthritis. *International Journal of Molecular Sciences*.

[44] Huang Z., Zhou M., Wang Q., Zhu M., Chen S., Li H. (2017). Mechanical and hypoxia stress can cause chondrocytes apoptosis through over- activation of endoplasmic reticulum stress. *Archives of Oral Biology*.

[45] Li H., Zhang X. Y., Wu T. J. (2013). Endoplasmic Reticulum Stress Regulates Rat Mandibular Cartilage Thinning under Compressive Mechanical Stress. *The Journal of Biological Chemistry*.

[46] Yang H., Wen Y., Zhang M. (2020). MTORC1 coordinates the autophagy and apoptosis signaling in articular chondrocytes in osteoarthritic temporomandibular joint. *Autophagy*.

[47] Hetz C. (2012). The unfolded protein response: controlling cell fate decisions under ER stress and beyond. *Nature Reviews Molecular Cell Biology*.

[48] Zhang M., Yang H., Lu L. (2017). Matrix replenishing by BMMSCs is beneficial for osteoarthritic temporomandibular joint cartilage. *Osteoarthritis and Cartilage*.

[49] Li Y., Li S., Luo Y., Liu Y., Yu N. (2017). LncRNA PVT1 regulates chondrocyte apoptosis in osteoarthritis by acting as a sponge for miR-488-3p. *DNA and Cell Biology*.

[50] Xu K., Meng Z., Xian X. M. (2020). LncRNA PVT1 induces chondrocyte apoptosis through upregulation of TNF-*α* in synoviocytes by sponging miR-211-3p. *Molecular and Cellular Probes*.

[51] van der Kraan P. M., van den Berg W. B. (2012). Chondrocyte hypertrophy and osteoarthritis: role in initiation and progression of cartilage degeneration?. *Osteoarthritis and Cartilage*.

[52] Kozhemyakina E., Lassar A. B., Zelzer E. (2015). A pathway to bone: signaling molecules and transcription factors involved in chondrocyte development and maturation. *Development*.

[53] Wei F., Zhou J., Wei X. (2012). Activation of Indian hedgehog promotes chondrocyte hypertrophy and upregulation of MMP-13 in human osteoarthritic cartilage. *Osteoarthritis and Cartilage*.

[54] Yang H., Zhang M., Liu Q. (2019). Inhibition of Ihh reverses temporomandibular joint osteoarthritis via a PTH1R signaling dependent mechanism. *International Journal of Molecular Sciences*.

[55] Zhang M., Yang H., Wan X. (2019). Prevention of injury-induced osteoarthritis in rodent temporomandibular joint by targeting chondrocyte CaSR. *Journal of Bone and Mineral Research*.

[56] Liu Q., Yang H. X., Wan X. H. (2018). Calcium-/calmodulin-dependent protein kinase II in occlusion-induced degenerative cartilage of rat mandibular condyle. *Journal of Oral Rehabilitation*.

[57] Mino-Oka A., Izawa T., Shinohara T. (2017). Roles of hypoxia inducible factor-1*α* in the temporomandibular joint. *Archives of Oral Biology*.

[58] Chen Y., Zhao B., Zhu Y., Zhao H., Ma C. (2019). HIF-1-VEGF-Notch mediates angiogenesis in temporomandibular joint osteoarthritis. *American Journal of Translational Research*.

[59] Wang Q.-Y., Dai J., Kuang B. (2012). Osteochondral angiogenesis in rat mandibular condyles with osteoarthritis-like changes. *Archives of Oral Biology*.

[60] Lan L., Jiang Y., Zhang W., Li T., Ying B., Zhu S. (2017). Expression of Notch signaling pathway during osteoarthritis in the temporomandibular joint. *Journal of Cranio-Maxillofacial Surgery*.

[61] Luo X., Jiang Y., Bi R., Jiang N., Zhu S. (2018). Inhibition of notch signaling pathway temporally postpones the cartilage degradation progress of temporomandibular joint arthritis in mice. *Journal of Cranio-Maxillofacial Surgery*.

[62] Ye T., He F., Lu L. (2020). The effect of oestrogen on mandibular condylar cartilage via hypoxia-inducible factor-2*α* during osteoarthritis development. *Bone*.

[63] Yang S., Kim J., Ryu J. H. (2010). Hypoxia-inducible factor-2*α* is a catabolic regulator of osteoarthritic cartilage destruction. *Nature Medicine*.

[64] Saito T., Fukai A., Mabuchi A. (2010). Transcriptional regulation of endochondral ossification by HIF-2*α* during skeletal growth and osteoarthritis development. *Nature Medicine*.

[65] Sperry M. M., Yu Y. H., Kartha S. (2020). Intra-articular etanercept attenuates pain and hypoxia from TMJ loading in the rat. *Journal of Orthopaedic Research*.

[66] Li W., Liu Y., Ding W., Long T., Shi J. (2017). Expression of hypoxia inducible factor-2 alpha in overloaded- stress induced destruction of mandibular condylar chondrocytes. *Archives of Oral Biology*.

[67] Ruscitto A. (2020). Evidence of vasculature and chondrocyte to osteoblast transdifferentiation in craniofacial synovial joints: implications for osteoarthritis diagnosis and therapy. *FASEB Journal*.

[68] Xiao J.-L., Meng J. H., Gan Y. H., Li Y. L., Zhou C. Y., Ma X. C. (2016). DNA methylation profiling in different phases of temporomandibular joint osteoarthritis in rats. *Archives of Oral Biology*.

[69] Ukita M., Matsushita K., Tamura M., Yamaguchi T. (2020). Histone H3K9 methylation is involved in temporomandibular joint osteoarthritis. *International Journal of Molecular Medicine*.

[70] Szabo L., Salzman J. (2016). Detecting circular RNAs: bioinformatic and experimental challenges. *Nature Reviews. Genetics*.

[71] Liu Q., Zhang X., Hu X. (2016). Circular RNA Related to the Chondrocyte ECM Regulates MMP13 Expression by Functioning as a MiR-136 'Sponge' in Human Cartilage Degradation. *Scientific Reports*.

[72] Wu Y., Zhang Y., Zhang Y., Wang J. J. (2017). CircRNA hsa_circ_0005105 upregulates NAMPT expression and promotes chondrocyte extracellular matrix degradation by sponging miR-26a. *Cell Biology International*.

[73] Hu Y., Zhu H., Bu L., He D. (2019). Expression profile of circular RNA s in TMJ osteoarthritis synovial tissues and potential functions of hsa_circ_0000448 with specific back-spliced junction. *American Journal of Translational Research*.

[74] Zhu H., Hu Y., Wang C., Zhang X., He D. (2020). CircGCN1L1 promotes synoviocyte proliferation and chondrocyte apoptosis by targeting miR-330-3p and TNF-*α* in TMJ osteoarthritis. *Cell Death & Disease*.

[75] Li W., Zhao S., Yang H. (2019). Potential novel prediction of TMJ-OA: miR-140-5p regulates inflammation through Smad/TGF-*β* signaling. *Frontiers in Pharmacology*.

[76] Horwitz E. M., le Blanc K., Dominici M. (2005). Clarification of the nomenclature for MSC: the International Society for Cellular Therapy position statement. *Cytotherapy*.

[77] Dominici M., le Blanc K., Mueller I. (2006). Minimal criteria for defining multipotent mesenchymal stromal cells. The International Society for Cellular Therapy position statement. *Cytotherapy*.

[78] Song Y., du H., Dai C. (2018). Human adipose-derived mesenchymal stem cells for osteoarthritis: a pilot study with long-term follow-up and repeated injections. *Regenerative Medicine*.

[79] Zellner J., Pattappa G., Koch M. (2017). Autologous mesenchymal stem cells or meniscal cells: what is the best cell source for regenerative meniscus treatment in an early osteoarthritis situation?. *Stem Cell Research & Therapy*.

[80] Vangsness C. T., Farr J., Boyd J., Dellaero D. T., Mills C. R., LeRoux-Williams M. (2014). Adult human mesenchymal stem cells delivered via intra-articular injection to the knee following partial medial meniscectomy. *The Journal of Bone and Joint Surgery*.

[81] Borakati A., Mafi R., Mafi P., Khan W. S. (2018). A systematic review and meta-analysis of clinical trials of mesenchymal stem cell therapy for cartilage repair. *Current Stem Cell Research & Therapy*.

[82] Fellows C. R., Matta C., Zakany R., Khan I. M., Mobasheri A. (2016). Adipose, bone marrow and synovial joint-derived mesenchymal stem cells for cartilage repair. *Frontiers in Genetics*.

[83] Lu L., Zhang X., Zhang M. (2015). RANTES and SDF-1 are keys in cell-based therapy of TMJ osteoarthritis. *Journal of Dental Research*.

[84] Lopa S., Colombini A., Moretti M., de Girolamo L. (2019). Injective mesenchymal stem cell-based treatments for knee osteoarthritis: from mechanisms of action to current clinical evidences. *Knee Surgery, Sports Traumatology, Arthroscopy*.

[85] Zhang J., Huang X., Wang H. (2015). The challenges and promises of allogeneic mesenchymal stem cells for use as a cell-based therapy. *Stem Cell Research & Therapy*.

[86] Chen K., Man C., Zhang B., Hu J., Zhu S. S. (2013). Effect of *in vitro* chondrogenic differentiation of autologous mesenchymal stem cells on cartilage and subchondral cancellous bone repair in osteoarthritis of temporomandibular joint. *International Journal of Oral and Maxillofacial Surgery*.

[87] van Buul G. M., Villafuertes E., Bos P. K. (2012). Mesenchymal stem cells secrete factors that inhibit inflammatory processes in short-term osteoarthritic synovium and cartilage explant culture. *Osteoarthritis and Cartilage*.

[88] Sato M., Uchida K., Nakajima H. (2012). Direct transplantation of mesenchymal stem cells into the knee joints of Hartley strain guinea pigs with spontaneous osteoarthritis. *Arthritis Research & Therapy*.

[89] Wakitani S., Nawata M., Tensho K., Okabe T., Machida H., Ohgushi H. (2007). Repair of articular cartilage defects in the patello-femoral joint with autologous bone marrow mesenchymal cell transplantation: three case reports involving nine defects in five knees. *Journal of Tissue Engineering and Regenerative Medicine*.

[90] Tarafder S., Koch A., Jun Y., Chou C., Awadallah M. R., Lee C. H. (2016). Micro-precise spatiotemporal delivery system embedded in 3D printing for complex tissue regeneration. *Biofabrication*.

[91] Legemate K., Tarafder S., Jun Y., Lee C. H. (2016). Engineering human TMJ discs with protein-releasing 3D-printed scaffolds. *Journal of Dental Research*.

[92] Zuk P. A., Zhu M., Ashjian P. (2002). Human adipose tissue is a source of multipotent stem cells. *Molecular Biology of the Cell*.

[93] Damia E., Chicharro D., Lopez S. (2018). Adipose-derived mesenchymal stem cells: are they a good therapeutic strategy for osteoarthritis?. *International Journal of Molecular Sciences*.

[94] Si Z., Wang X., Sun C. (2019). Adipose-derived stem cells: sources, potency, and implications for regenerative therapies. *Biomedicine & Pharmacotherapy*.

[95] Mazini L., Rochette L., Amine M., Malka G. (2019). Regenerative capacity of adipose derived stem cells (ADSCs), comparison with mesenchymal stem cells (MSCs). *International Journal of Molecular Sciences*.

[96] Aust L., Devlin B., Foster S. J. (2004). Yield of human adipose-derived adult stem cells from liposuction aspirates. *Cytotherapy*.

[97] Mirsaidi A., Kleinhans K. N., Rimann M. (2012). Telomere length, telomerase activity and osteogenic differentiation are maintained in adipose-derived stromal cells from senile osteoporotic SAMP6 mice. *Journal of Tissue Engineering and Regenerative Medicine*.

[98] Dufrane D. (2017). Impact of age on human adipose stem cells for bone tissue engineering. *Cell Transplantation*.

[99] Mäenpää K., Ellä V., Mauno J. (2010). Use of adipose stem cells and polylactide discs for tissue engineering of the temporomandibular joint disc. *Journal of The Royal Society Interface*.

[100] Rivera-Izquierdo M., Cabeza L., Láinez-Ramos-Bossini A. (2019). An updated review of adipose derived-mesenchymal stem cells and their applications in musculoskeletal disorders. *Expert Opinion on Biological Therapy*.

[101] Lee W.-S., Kim H. J., Kim K. I., Kim G. B., Jin W. (2019). Intra-articular injection of autologous adipose tissue-derived mesenchymal stem cells for the treatment of knee osteoarthritis: a phase IIb, randomized, placebo-controlled clinical trial. *Stem Cells Translational Medicine*.

[102] Jo C. H., Lee Y. G., Shin W. H. (2014). Intra-articular injection of mesenchymal stem cells for the treatment of osteoarthritis of the knee: a proof-of-concept clinical trial. *Stem Cells*.

[103] Pers Y.-M., Rackwitz L., Ferreira R. (2016). Adipose mesenchymal stromal cell-based therapy for severe osteoarthritis of the knee: a phase I dose-escalation trial. *Stem Cells Translational Medicine*.

[104] Ahtiainen K., Mauno J., Ellä V. (2013). Autologous adipose stem cells and polylactide discs in the replacement of the rabbit temporomandibular joint disc. *Journal of The Royal Society Interface*.

[105] Carboni A., Amodeo G., Perugini M., Arangio P., Orsini R., Scopelliti D. (2019). Temporomandibular disorders clinical and anatomical outcomes after fat-derived stem cells injection. *The Journal of Craniofacial Surgery*.

[106] Kim D. W., Staples M., Shinozuka K., Pantcheva P., Kang S. D., Borlongan C. (2013). Wharton’s jelly-derived mesenchymal stem cells: phenotypic characterization and optimizing their therapeutic potential for clinical applications. *International Journal of Molecular Sciences*.

[107] Baksh D., Yao R., Tuan R. S. (2007). Comparison of proliferative and multilineage differentiation potential of human mesenchymal stem cells derived from umbilical cord and bone marrow. *Stem Cells*.

[108] Ranjbaran H., Abediankenari S., Mohammadi M. (2018). Wharton’s jelly derived-mesenchymal stem cells: isolation and characterization. *Acta Medica Iranica*.

[109] Ding D. C., Chang Y. H., Shyu W. C., Lin S. Z. (2015). Human umbilical cord mesenchymal stem cells: a new era for stem cell therapy. *Cell Transplantation*.

[110] Richardson S. M., Kalamegam G., Pushparaj P. N. (2016). Mesenchymal stem cells in regenerative medicine: focus on articular cartilage and intervertebral disc regeneration. *Methods*.

[111] Klontzas M. E., Kenanidis E. I., Heliotis M., Tsiridis E., Mantalaris A. (2015). Bone and cartilage regeneration with the use of umbilical cord mesenchymal stem cells. *Expert Opinion on Biological Therapy*.

[112] Troyer D. L., Weiss M. L. (2008). Wharton’s jelly-derived cells are a primitive stromal cell population. *Stem Cells*.

[113] Wang L., Tran I., Seshareddy K., Weiss M. L., Detamore M. S. (2009). A comparison of human bone marrow-derived mesenchymal stem cells and human umbilical cord-derived mesenchymal stromal cells for cartilage tissue engineering. *Tissue Engineering Part A*.

[114] Bailey M. M., Wang L., Bode C. J., Mitchell K. E., Detamore M. S. (2007). A comparison of human umbilical cord matrix stem cells and temporomandibular joint condylar chondrocytes for tissue engineering temporomandibular joint condylar cartilage. *Tissue Engineering*.

[115] Wang L., Seshareddy K., Weiss M. L., Detamore M. S. (2009). Effect of initial seeding density on human umbilical cord mesenchymal stromal cells for fibrocartilage tissue engineering. *Tissue Engineering Part A*.

[116] Kim H., Yang G., Park J., Choi J., Kang E., Lee B. K. (2019). Therapeutic effect of mesenchymal stem cells derived from human umbilical cord in rabbit temporomandibular joint model of osteoarthritis. *Scientific Reports*.

[117] Jeon H. J., Yoon K. A., An E. S. (2020). Therapeutic effects of human umbilical cord blood-derived mesenchymal stem cells combined with cartilage acellular matrix mediated via bone morphogenic protein 6 in a rabbit model of articular cruciate ligament transection. *Stem Cell Reviews and Reports*.

[118] Kim S. E., Pozzi A., Yeh J. C. (2019). Intra-articular umbilical cord derived mesenchymal stem cell therapy for chronic elbow osteoarthritis in dogs: a double-blinded, Placebo-Controlled Clinical Trial. *Frontiers in Veterinary Science*.

[119] Gronthos S., Mankani M., Brahim J., Robey P. G., Shi S. (2000). Postnatal human dental pulp stem cells (DPSCs) in vitro and in vivo. *Proceedings of the National Academy of Sciences of the United States of America*.

[120] Saito M. T., Silvério K. G., Casati M. Z., Sallum E. A., Nociti FH Jr (2015). Tooth-derived stem cells: update and perspectives. *World Journal of Stem Cells*.

[121] Sharpe P. T. (2016). Dental mesenchymal stem cells. *Development*.

[122] Park Y. J., Cha S., Park Y. S. (2016). Regenerative applications using tooth derived stem cells in other than tooth regeneration: a literature review. *Stem Cells International*.

[123] Lei M., Li K., Li B., Gao L. N., Chen F. M., Jin Y. (2014). Mesenchymal stem cell characteristics of dental pulp and periodontal ligament stem cells after *in vivo* transplantation. *Biomaterials*.

[124] Kunimatsu R., Nakajima K., Awada T. (2018). Comparative characterization of stem cells from human exfoliated deciduous teeth, dental pulp, and bone marrow-derived mesenchymal stem cells. *Biochemical and Biophysical Research Communications*.

[125] Mata M., Milian L., Oliver M. (2017). In vivo articular cartilage regeneration using human dental pulp stem cells cultured in an alginate scaffold: a preliminary study. *Stem Cells International*.

[126] Garland C. B., Pomerantz J. H. (2012). Regenerative strategies for craniofacial disorders. *Frontiers in Physiology*.

[127] Tatullo M., Marrelli M., Shakesheff K. M., White L. J. (2015). Dental pulp stem cells: function, isolation and applications in regenerative medicine. *Journal of Tissue Engineering and Regenerative Medicine*.

[128] Yanasse R. H., de Lábio R. W., Marques L. (2019). Xenotransplantation of human dental pulp stem cells in platelet-rich plasma for the treatment of full-thickness articular cartilage defects in a rabbit model. *Experimental and Therapeutic Medicine*.

[129] Dai J., Wang J., Lu J. (2012). The effect of co-culturing costal chondrocytes and dental pulp stem cells combined with exogenous FGF9 protein on chondrogenesis and ossification in engineered cartilage. *Biomaterials*.

[130] Bousnaki M., Bakopoulou A., Papadogianni D. (2018). Fibro/chondrogenic differentiation of dental stem cells into chitosan/alginate scaffolds towards temporomandibular joint disc regeneration. *Journal of Materials Science. Materials in Medicine*.

[131] Zhang J., Guo F., Mi J., Zhang Z. (2014). Periodontal ligament mesenchymal stromal cells increase proliferation and glycosaminoglycans formation of temporomandibular joint derived fibrochondrocytes. *BioMed Research International*.

[132] Ogasawara N., Kano F., Hashimoto N. (2020). Factors secreted from dental pulp stem cells show multifaceted benefits for treating experimental temporomandibular joint osteoarthritis. *Osteoarthritis and Cartilage*.

[133] Wu L., Leijten J. C. H., Georgi N., Post J. N., van Blitterswijk C. A., Karperien M. (2011). Trophic effects of mesenchymal stem cells increase chondrocyte proliferation and matrix formation. *Tissue Engineering Part A*.

[134] Campbell K., Naire S., Kuiper J. H. (2019). A mathematical model of cartilage regeneration after chondrocyte and stem cell implantation – II: the effects of co-implantation. *Journal of Tissue Engineering*.

[135] Acharya C., Adesida A., Zajac P. (2012). Enhanced chondrocyte proliferation and mesenchymal stromal cells chondrogenesis in coculture pellets mediate improved cartilage formation. *Journal of Cellular Physiology*.

[136] Wu L., Prins H. J., Helder M. N., van Blitterswijk C. A., Karperien M. (2012). Trophic effects of mesenchymal stem cells in chondrocyte co-cultures are independent of culture conditions and cell sources. *Tissue Engineering Part A*.

[137] Koyama N., Okubo Y., Nakao K., Osawa K., Fujimura K., Bessho K. (2011). Pluripotency of mesenchymal cells derived from synovial fluid in patients with temporomandibular joint disorder. *Life Sciences*.

[138] Sun Y. P., Zheng Y. H., Liu W. J., Zheng Y. L., Zhang Z. G. (2014). Synovium fragment-derived cells exhibit characteristics similar to those of dissociated multipotent cells in synovial fluid of the temporomandibular joint. *PLoS One*.

[139] Shen W., Chen J., Zhu T. (2014). Intra-articular injection of human meniscus stem/progenitor cells promotes meniscus regeneration and ameliorates osteoarthritis through stromal cell-derived factor-1/CXCR4-mediated homing. *Stem Cells Translational Medicine*.

[140] Embree M. C., Chen M., Pylawka S. (2016). Exploiting endogenous fibrocartilage stem cells to regenerate cartilage and repair joint injury. *Nature Communications*.

[141] McGonagle D., Baboolal T. G., Jones E. (2017). Native joint-resident mesenchymal stem cells for cartilage repair in osteoarthritis. *Nature Reviews Rheumatology*.

[142] Zhang S., Hu B., Liu W. (2020). Articular cartilage regeneration: the role of endogenous mesenchymal stem/progenitor cell recruitment and migration. *Seminars in Arthritis and Rheumatism*.

[143] Ando W., Kutcher J. J., Krawetz R. (2014). Clonal analysis of synovial fluid stem cells to characterize and identify stable mesenchymal stromal cell/mesenchymal progenitor cell phenotypes in a porcine model: a cell source with enhanced commitment to the chondrogenic lineage. *Cytotherapy*.

[144] Jones B. A., Pei M. (2012). Synovium-derived stem cells: a tissue-specific stem cell for cartilage engineering and regeneration. *Tissue Engineering Part B, Reviews*.

[145] Lin Y., Umebayashi M., Abdallah M. N. (2019). Combination of polyetherketoneketone scaffold and human mesenchymal stem cells from temporomandibular joint synovial fluid enhances bone regeneration. *Scientific Reports*.

[146] Horie M., Sekiya I., Muneta T. (2009). Intra-articular injected synovial stem cells differentiate into meniscal cells directly and promote meniscal regeneration without mobilization to distant organs in rat massive meniscal defect. *Stem Cells*.

[147] Liang Y., Idrees E., Szojka A. R. A. (2018). Chondrogenic differentiation of synovial fluid mesenchymal stem cells on human meniscus-derived decellularized matrix requires exogenous growth factors. *Acta Biomaterialia*.

[148] Hatsushika D., Muneta T., Nakamura T. (2014). Repetitive allogeneic intraarticular injections of synovial mesenchymal stem cells promote meniscus regeneration in a porcine massive meniscus defect model. *Osteoarthritis and Cartilage*.

[149] Wu Y., Gong Z., Li J., Meng Q., Fang W., Long X. (2014). The pilot study of fibrin with temporomandibular joint derived synovial stem cells in repairing TMJ disc perforation. *BioMed Research International*.

[150] Jones E. A., Crawford A., English A. (2008). Synovial fluid mesenchymal stem cells in health and early osteoarthritis: detection and functional evaluation at the single-cell level. *Arthritis and Rheumatism*.

[151] al-Moraissi E. A., Wolford L. M., Ellis E., Neff A. (2020). The hierarchy of different treatments for arthrogenous temporomandibular disorders: a network meta-analysis of randomized clinical trials. *Journal of Cranio-Maxillo-Facial Surgery*.

[152] Baboolal T. G., Khalil-Khan A., Theodorides A. A., Wall O., Jones E., McGonagle D. (2018). A novel arthroscopic technique for intraoperative mobilization of synovial mesenchymal stem cells. *The American Journal of Sports Medicine*.

[153] Liu W., Sun Y., He Y. (2017). IL-1*β* impedes the chondrogenic differentiation of synovial fluid mesenchymal stem cells in the human temporomandibular joint. *International Journal of Molecular Medicine*.

[154] Heldens G. T., Blaney Davidson E. N., Vitters E. L. (2012). Catabolic factors and osteoarthritis-conditioned medium inhibit chondrogenesis of human mesenchymal stem cells. *Tissue Engineering Part A*.

[155] Nishimura M., Segami N., Kaneyama K., Suzuki T., Miyamaru M. (2002). Proinflammatory cytokines and arthroscopic findings of patients withinternal derangement and osteoarthritis of the temporomandibular joint. *The British Journal of Oral & Maxillofacial Surgery*.

[156] Liao W., Sun J., Liu W. (2019). HDAC10 upregulation contributes to interleukin 1*β*-mediated inflammatory activation of synovium-derived mesenchymal stem cells in temporomandibular joint. *Journal of Cellular Physiology*.

[157] Sun J., Liao W., Su K. (2020). Suberoylanilide hydroxamic acid attenuates interleukin-1*β*-induced interleukin-6 upregulation by inhibiting the microtubule affinity-regulating kinase 4/nuclear factor-*κ*B pathway in synovium-derived mesenchymal stem cells from the temporomandibular joint. *Inflammation*.

[158] Chan B. Y., Fuller E. S., Russell A. K. (2011). Increased chondrocyte sclerostin may protect against cartilage degradation in osteoarthritis. *Osteoarthritis and Cartilage*.

[159] Bouaziz W., Funck-Brentano T., Lin H. (2015). Loss of sclerostin promotes osteoarthritis in mice via *β*-catenin-dependent and -independent Wnt pathways. *Arthritis Research & Therapy*.

[160] Théry C., Zitvogel L., Amigorena S. (2002). Exosomes: composition, biogenesis and function. *Nature Reviews Immunology*.

[161] Yáñez-Mó M., Siljander P. R. M., Andreu Z. (2015). Biological properties of extracellular vesicles and their physiological functions. *Journal of Extracellular Vesicles*.

[162] Mianehsaz E., Mirzaei H. R., Mahjoubin-Tehran M. (2019). Mesenchymal stem cell-derived exosomes: a new therapeutic approach to osteoarthritis?. *Stem Cell Research & Therapy*.

[163] Baglio S. R., Rooijers K., Koppers-Lalic D. (2015). Human bone marrow- and adipose-mesenchymal stem cells secrete exosomes enriched in distinctive miRNA and tRNA species. *Stem Cell Research & Therapy*.

[164] Qin Y., Wang L., Gao Z., Chen G., Zhang C. (2016). Bone marrow stromal/stem cell-derived extracellular vesicles regulate osteoblast activity and differentiation *in vitro* and promote bone regeneration *in vivo*. *Scientific Reports*.

[165] Zhou Y., Xu H., Xu W. (2013). Exosomes released by human umbilical cord mesenchymal stem cells protect against cisplatin-induced renal oxidative stress and apoptosis in vivo and in vitro. *Stem Cell Research & Therapy*.

[166] Wang Y., Yu D., Liu Z. (2017). Exosomes from embryonic mesenchymal stem cells alleviate osteoarthritis through balancing synthesis and degradation of cartilage extracellular matrix. *Stem Cell Research & Therapy*.

[167] Qi X., Zhang J., Yuan H. (2016). Exosomes secreted by human-induced pluripotent stem cell-derived mesenchymal stem cells repair critical-sized bone defects through enhanced angiogenesis and osteogenesis in osteoporotic rats. *International Journal of Biological Sciences*.

[168] Lai R. C., Arslan F., Lee M. M. (2010). Exosome secreted by MSC reduces myocardial ischemia/reperfusion injury. *Stem Cell Research*.

[169] Zhang S., Chu W. C., Lai R. C., Lim S. K., Hui J. H. P., Toh W. S. (2016). Exosomes derived from human embryonic mesenchymal stem cells promote osteochondral regeneration. *Osteoarthritis and Cartilage*.

[170] Cosenza S., Ruiz M., Toupet K., Jorgensen C., Noël D. (2017). Mesenchymal stem cells derived exosomes and microparticles protect cartilage and bone from degradation in osteoarthritis. *Scientific Reports*.

[171] Ding J., Chen B., Lv T. (2016). Bone marrow mesenchymal stem cell-based engineered cartilage ameliorates polyglycolic acid/polylactic acid scaffold-induced inflammation through M2 polarization of macrophages in a pig model. *Stem Cells Translational Medicine*.

[172] Fahy N., de Vries-van Melle M. L., Lehmann J. (2014). Human osteoarthritic synovium impacts chondrogenic differentiation of mesenchymal stem cells via macrophage polarisation state. *Osteoarthritis and Cartilage*.

[173] Zhang S., Chuah S. J., Lai R. C., Hui J. H. P., Lim S. K., Toh W. S. (2018). MSC exosomes mediate cartilage repair by enhancing proliferation, attenuating apoptosis and modulating immune reactivity. *Biomaterials*.

[174] Luo P., Jiang C., Ji P., Wang M., Xu J. (2019). Exosomes of stem cells from human exfoliated deciduous teeth as an anti-inflammatory agent in temporomandibular joint chondrocytes via miR-100-5p/mTOR. *Stem Cell Research & Therapy*.

[175] Anz A. (2016). Current and future stem cell regulation: a call to action. *American Journal of Orthopedics*.

[176] di Matteo B., Vandenbulcke F., Vitale N. D. (2019). Minimally manipulated mesenchymal stem cells for the treatment of knee osteoarthritis: a systematic review of clinical evidence. *Stem Cells International*.

[177] Chahla J., Mannava S., Cinque M. E., Geeslin A. G., Codina D., LaPrade R. F. (2017). Bone marrow aspirate concentrate harvesting and processing technique. *Arthroscopy Techniques*.

[178] Mautner K., Bowers R., Easley K., Fausel Z., Robinson R. (2019). Functional outcomes following microfragmented adipose tissue versus bone marrow aspirate concentrate injections for symptomatic knee osteoarthritis. *Stem Cells Translational Medicine*.

[179] Anz A. W., Hubbard R., Rendos N. K., Everts P. A., Andrews J. R., Hackel J. G. (2020). Bone marrow aspirate concentrate is equivalent to platelet-rich plasma for the treatment of knee osteoarthritis at 1 year: a prospective, randomized trial. *Orthopaedic Journal of Sports Medicine*.

[180] Rodriguez-Fontan F., Piuzzi N. S., Kraeutler M. J., Pascual-Garrido C. (2018). Early clinical outcomes of intra-articular injections of bone marrow aspirate concentrate for the treatment of early osteoarthritis of the hip and knee: a cohort study. *PM & R*.

[181] Shapiro S. A., Kazmerchak S. E., Heckman M. G., Zubair A. C., O’Connor M. I. (2017). A prospective, single-blind, placebo-controlled trial of bone marrow aspirate concentrate for knee osteoarthritis. *The American Journal of Sports Medicine*.

[182] Shapiro S. A., Arthurs J. R., Heckman M. G. (2018). Quantitative T2 MRI mapping and 12-month follow-up in a randomized, blinded, placebo controlled trial of bone marrow aspiration and concentration for osteoarthritis of the knees. *Cartilage*.

[183] Tonnard P., Verpaele A., Peeters G., Hamdi M., Cornelissen M., Declercq H. (2013). Nanofat grafting: basic research and clinical applications. *Plastic and Reconstructive Surgery*.

[184] Pak J. (2011). Regeneration of human bones in hip osteonecrosis and human cartilage in knee osteoarthritis with autologous adipose-tissue-derived stem cells: a case series. *Journal of Medical Case Reports*.

[185] Mehranfar S., Abdi Rad I., Mostafavi E., Akbarzadeh A. (2019). The use of stromal vascular fraction (SVF), platelet-rich plasma (PRP) and stem cells in the treatment of osteoarthritis: an overview of clinical trials. *Artificial Cells, Nanomedicine, and Biotechnology*.

[186] Roato I., Belisario D. C., Compagno M. (2019). Concentrated adipose tissue infusion for the treatment of knee osteoarthritis: clinical and histological observations. *International Orthopaedics*.

[187] Borić I., Hudetz D., Rod E. (2019). A 24-month follow-up study of the effect of intra-articular injection of autologous microfragmented fat tissue on proteoglycan synthesis in patients with knee osteoarthritis. *Genes*.

[188] Mahmmood V. H., Shihab S. M. (2019). Assessment of therapeutic effect of intra-articular nanofat injection for temporomandibular disorders. *The Journal of Craniofacial Surgery*.

[189] Kim H., Lee B. K. (2020). Anti-inflammatory effect of adipose-derived stromal vascular fraction on osteoarthritic temporomandibular joint synoviocytes. *Tissue Eng Regen Med*.

[190] Inoue S., Popp F. C., Koehl G. E. (2006). Immunomodulatory effects of mesenchymal stem cells in a rat organ transplant model. *Transplantation*.

[191] Waterman R. S., Tomchuck S. L., Henkle S. L., Betancourt A. M. (2010). A new mesenchymal stem cell (MSC) paradigm: polarization into a pro-inflammatory MSC1 or an immunosuppressive MSC2 phenotype. *PLoS One*.

[192] Lee S. Y., Lee S. H., Na H. S. (2018). The therapeutic effect of STAT3 signaling-suppressed MSC on pain and articular cartilage damage in a rat model of monosodium iodoacetate-induced osteoarthritis. *Frontiers in Immunology*.

[193] Reiner A. T., Witwer K. W., van Balkom B. W. M. (2017). Concise review: developing best-practice models for the therapeutic use of extracellular vesicles. *Stem Cells Translational Medicine*.

